# The impact of NK1 receptor antagonist selection on chemotherapy-related adverse events: a retrospective study of adverse events in paclitaxel or nab-paclitaxel and carboplatin combination therapy with fosnetupitant and aprepitant use

**DOI:** 10.3389/fphar.2026.1712949

**Published:** 2026-05-11

**Authors:** Kenta Yamaoka, Saki Kajita, Manabu Kume, Masaki Hirabatake, Yuki Sato, Tohru Hashida, Ryo Tachikawa, Nobuyuki Muroi

**Affiliations:** 1 Department of Pharmacy, Kobe City Medical Center General Hospital, Kobe, Japan; 2 Department of Pharmacy, Kobe City Medical Center West Hospital, Kobe, Japan; 3 Department of Respiratory Medicine, Kobe City Medical Center General Hospital, Kobe, Japan

**Keywords:** aprepitant, drug interaction, fosnetupitant, neutropenia, non-small cell lung cancer, paclitaxel

## Abstract

Netupitant (NTP), the active form of fosnetupitant (FosNTP), a novel NK1 receptor antagonist, has been reported to possess a longer half-life than conventional drugs and demonstrates favorable antiemetic effects in the delayed phase. However, NTP has been reported to exert an inhibitory effect on CYP3A4, similar to conventional NK1 receptor antagonists. Paclitaxel (PTX), which is utilized in the treatment of non-small cell lung cancer, undergoes metabolism via CYP3A4. The enhancement of myelosuppression resulting from this interaction may necessitate dose reduction or withdrawal of anticancer drugs, which may compromise treatment continuation. While it has been reported that aprepitant (APR) does not influence the incidence of adverse events associated with anticancer drugs metabolized via CYP3A4, reports on FosNTP remain limited. Therefore, this study retrospectively examined the differences in the incidence of myelosuppression as an adverse event in patients with non-small cell lung cancer treated with FosNTP and APR. Patients treated with PTX or nanoparticle albumin-bound paclitaxel (nab-PTX) combined with platinum-based anticancer regimens for non-small cell lung cancer between January 2019 and September 2024 were included in this study. Patients receiving FosNTP and APR as antiemetic agents were stratified into groups, and the incidence of grade ≥3 myelosuppression served as the primary endpoint. Statistical analysis was performed using the chi-square test, with statistical significance established at P < 0.05. The incidence of grade ≥3 neutropenia was 15/31 in the FosNTP group and 18/68 in the APR group among PTX-treated patients, demonstrating a significantly higher incidence in the FosNTP group (48.4% vs. 26.5%; p = 0.019). The extended half-life of NTP compared to that of APR may have contributed to prolonged CYP3A4 inhibition and subsequent inhibition of PTX metabolism. Conversely, among nab-PTX users, 10/49 patients in the FosNTP group and 8/53 patients in the APR group developed grade ≥3 neutropenia, with no significant difference observed (20.4% vs. 17.0%; p = 0.182). This study suggests that the use of FosNTP in PTX-containing regimens may lead to a higher incidence of grade ≥3 neutropenia compared to APR, emphasizing the importance of vigilant monitoring throughout the treatment period.

## 1 Introduction

In cancer chemotherapy, nausea and vomiting are two of the most distressing adverse effects that patients experience, potentially compromising treatment continuation (Gupta et al., 2021). Antiemetic therapy constitutes an extremely important supportive intervention for maintaining the cancer chemotherapy intensity and ensuring treatment completion. The combination of conventional platinum-based chemotherapy with third-generation cytotoxic chemotherapy has demonstrated high efficacy against non-small cell lung cancer (Schiller et al., 2002). However, nausea and vomiting are typical adverse events associated with platinum-based anticancer drugs. When carboplatin (CBDCA)-containing chemotherapy is administered, antiemetic therapy corresponding to the high emetogenic risk category is recommended by guidelines from various academic societies (Herrstedt et al., 2017; Hesketh et al., 2020).

Generally, combination therapy comprising NK1 receptor antagonists, 5-HT3 receptor antagonists, and dexamethasone is recommended as antiemetic therapy for highly emetogenic-risk anticancer drugs (Herrstedt et al., 2017; Hesketh et al., 2020). Recently, the antiemetic efficacy of olanzapine has been reported, with evidence suggesting that enhanced antiemetic effects can be anticipated when olanzapine is added to these three agents (Chiu et al., 2016; Navari et al., 2016; Hashimoto et al., 2020). However, anticancer drug-induced nausea and vomiting have been documented to persist into the late-onset phase, extending to 120 h post-administration (Chow et al., 2023; Tamura et al., 2015). Currently, effective treatments for late-onset nausea and vomiting remain limited, prompting various ongoing investigations (Chow et al., 2023).

Aprepitant (APR), a widely utilized NK1 receptor antagonist, exerts moderate inhibition of cytochrome P450 (CYP) 3A4 (Sanchez et al., 2004). CYP3A4 mediates the metabolism of numerous drugs, including paclitaxel (PTX) and other anticancer agents. Previous reports have demonstrated that concomitant administration of APR with docetaxel, vinorelbine, cyclophosphamide, and cabazitaxel, which undergo CYP3A4-mediated metabolism, neither affected pharmacokinetics nor increased adverse events (Bubalo et al., 2012; Kaneta et al., 2014; Loos et al., 2007; Nygren et al., 2005; Sarantopoulos et al., 2014; Walko et al., 2012). Additionally, fosaprepitant (FosAPR), a prodrug formulation of APR designed for intravenous infusion, requires solitary administration due to the high incidence of injection site pain and the potential for drug incompatibilities (Nishibe-Toyosato et al., 2024). This requirement increases the burden on healthcare professionals, necessitating the selection of larger vessels, prolongation of administration time, and frequent infusion bottle changes when FosAPR is administered alone.

Recently, a novel NK1 receptor antagonist, fosnetupitant (FosNTP), received approval for use in Japan. FosNTP functions as a prodrug of netupitant (NTP). NTP exhibits a longer half-life than APR and FosAPR, with its antiemetic effect reportedly persisting against late-onset symptoms (Hata et al., 2022; Hesketh et al., 2014; Sugawara et al., 2019; Zhang et al., 2018). Furthermore, FosNTP has been documented to not only reduce injection site pain frequency compared to FosAPR but also demonstrate compatibility with a greater number of medications (Kubota et al., 2025). These advantages benefit both patients and healthcare professionals, facilitating widespread adoption as facilities transition from conventional NK1 antagonists. While FosNTP, similar to APR and FosAPR, functions as a moderate inhibitor of CYP3A4, reports regarding the frequency of adverse events associated with its concomitant use with CYP3A4-metabolized anticancer drugs remain limited (Abe et al., 2023; Deb and Hopefl, 2023; Lanzarotti and Rossi, 2013). In the CONSOLE trial, neutropenia was reported as an adverse event in 0% of patients receiving FosNTP, compared with 3.8% in those treated with FosAPR. Nevertheless, as the published report did not specify the detailed treatment regimens, the contextual interpretation of these results remains limited. Further clarification of regimen-specific factors will be essential to fully elucidate the comparative safety profiles of these agents (Hata et al., 2022).

This study focused on patients treated with PTX or nanoparticle albumin-bound paclitaxel (nab-PTX) plus CBDCA for stage IV non-small cell lung cancer and compared the incidence of severe grade ≥3 myelosuppression between APR and FosNTP administration. The study aimed to elucidate the impact of NK1 receptor antagonist selection on chemotherapy-related adverse events, not only to enhance the quality of life of patients but also to facilitate appropriate antiemetic therapy selection by preventing anticancer drug withdrawal, dose reduction, or treatment postponement.

## 2 Methods

### 2.1 Collection of patient information and outcome assessment

This study included patients who received PTX or nab-PTX in combination with CBDCA for stage IV non-small cell lung cancer from January 2019 to September 2024 at Kobe City Medical Center Chuo Municipal Hospital and utilized FosNTP or APR as antiemetic therapy. The administration schedules for PTX + CBDCA and nab-PTX + CBDCA at our institution are presented in Supplementary Tables S1, S2. The exclusion criteria comprised patients who received additional NK1 receptor antagonists during the treatment period, those who initiated treatment at another hospital, those who discontinued chemotherapy during the first cycle, those with CBDCA doses corresponding to an area under the drug concentration-time curve <4, those who received peg-G-CSF for primary prevention of febrile neutropenia, those who received drugs with strong or moderate CYP3A4 inhibition or induction at treatment initiation, and those younger than 20 years of age. The primary endpoint was the differential incidence of grade ≥3 myelosuppression between antiemetic regimens throughout the treatment period. Secondary endpoints included the differential incidence of grade ≥3 myelosuppression after the first cycle, the differential incidence of liver dysfunction throughout the treatment period, and the identification of risk factors associated with grade ≥3 neutropenia and anemia.

### 2.2 Data collection and evaluation

To evaluate the occurrence of grade ≥3 adverse effects, the following patient information was retrospectively extracted from electronic medical records: age, sex, body weight, body surface area, anticancer drug dosage, concomitant medications, prior therapy, serum albumin level, white blood cell count, total neutrophil count, hemoglobin level (from treatment initiation to termination), platelet count, aspartate aminotransferase (AST), alanine aminotransferase, and total bilirubin. Grade classification was performed according to the Common Terminology Criteria for Adverse Events (CTCAE) version 5.0. For patients who transitioned to different NK1 receptor antagonists during treatment, the evaluation period encompassed only the duration until the transition occurred. The observation period concluded on 31 December 2024, regardless of the ongoing treatment status.

### 2.3 Statistical analysis

**FIGURE 1:**
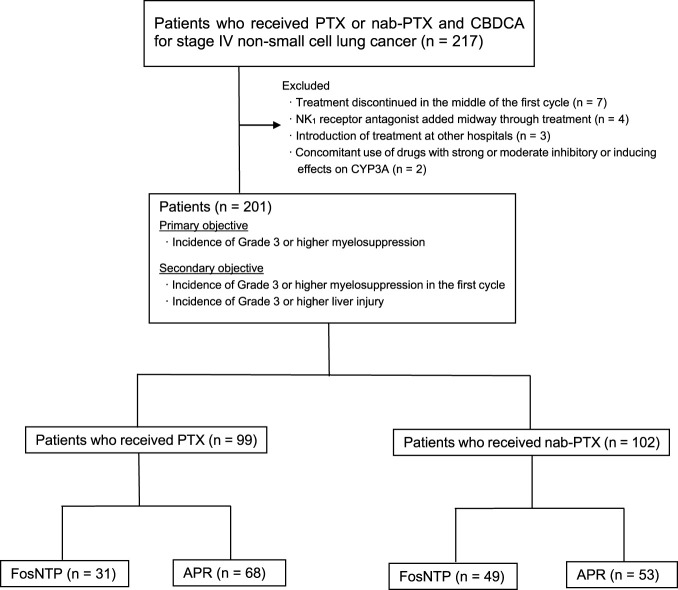
Study diagram. PTX, paclitaxel; nab-PTX, nab-paclitaxel; FosNTP, fosnetupitant; APR, aprepitant; CBDCA, carboplatin.

Patients treated with PTX and nab-PTX were stratified into two groups: those receiving FosNTP and those receiving APR. The specific patient groupings are illustrated in Figure 1. The incidence of myelosuppression and liver dysfunction was assessed at the end of the first cycle and throughout the entire treatment period for each group, with inter-group comparisons of the adverse effect incidence conducted for patients receiving the same chemotherapeutic agent. Categorical data are presented as patient numbers and percentages (%), with Fisher’s exact test employed for between-group comparisons. Continuous data are expressed as medians with interquartile ranges. Given the small number of patients initially treated with FosNTP after its approval and the resulting potential imbalance in baseline characteristics, multivariate logistic regression analyses were conducted to identify independent predictors of grade ≥ 3 neutropenia and anemia. Candidate covariates were selected *a priori* based on previously published evidence regarding hematologic toxicity (Ito et al., 2021; Hagiwara et al., 2022; Tsushima et al., 2020). Grade ≥ 3 thrombocytopenia and hepatic dysfunction were not included in the models because of their very low event rates. A two-sided p value < 0.05 was considered statistically significant. All statistical analyses were performed using JMP PRO®18.0.0 (SAS Institute, Cary, NC, USA).

### 2.4 Ethical considerations

The study was conducted in accordance with the principles of the Declaration of Helsinki. An opt-out method was used to use participant data for research purposes. The study protocol and consent procedures were reviewed and approved by the Ethics Committee of the Kobe City Medical Center Central Municipal Hospital (approval number: zn250702; approval date: 13 June 2025).

## 3 Results

### 3.1 Patient baseline characteristics

Between January 2019 and September 2024, 217 patients received antiemetic therapy with FosNTP or APR for PTX or nab-PTX and CBDCA combination therapy for non-small cell lung cancer, with 201 patients ultimately enrolled in the study. The enrolled patients were stratified into four groups: PTX-FosNTP (n = 31), PTX-APR (n = 68), nab-PTX-FosNTP (n = 49), and nab-PTX-APR (n = 53). Patient baseline characteristics are presented in Table 1. The study population comprised 156 males (77.6%) and 45 females (22.4%). Among enrolled patients, 109 (54.2%) had adenocarcinoma, 75 (37.3%) had squamous cell carcinoma, and 37 (18.4%) were epidermal growth factor receptor mutation-positive. During CBDCA + PTX or nab-PTX treatment, 57 patients (28.4%) received bevacizumab, and 78 patients (38.8%) were administered immune checkpoint inhibitors. However, baseline differences in hemoglobin levels between the groups may have confounded these findings and cannot be fully ruled out.

**TABLE 1:** Baseline patient characteristics.

Characteristic	PTX (n = 99)			Nab-PTX (n = 102)		
	FosNTP (n = 31)	APR (n = 68)	p- value	FosNTP (n = 49)	APR (n = 53)	p-value
Age (years), median (IQR)	72 (58–77)	70 (63–74)	0.383	74 (69–79)	70 (68–76)	0.296
Sex, no. (%)	​	​	0.631	​	​	0.447
Male	24 (77.4%)	49 (72.1%)	​	38 (77.6%)	45 (84.9%)	​
Female	7 (22.6%)	19 (27.9%)	​	11 (22.4%)	8 (15.1%)	​
Weight (kg), median (IQR)	60.5 (54.3–73.1)	62.3 (56.2–67.6)	0.868	62.5 (52.2–68.4)	59.4 (51.8–68.9)	0.546
Body surface area (m^2^), median (IQR)	1.66 (1.56–1.85)	1.69 (1.59–1.77)	0.514	1.66 (1.51–1.74)	1.66 (1.54–1.80)	0.280
Alb (g/dL), median (IQR)	3.7 (3.2–4.1)	3.8 (3.4–4.1)	0.182	3.4 (3.0–3.8)	3.7 (3.2–4.0)	0.170
AST (U/L), median (IQR)	22 (17–29)	21 (17–27)	0.700	20 (16–25)	18 (16–24)	0.634
ALT (U/L), median (IQR)	19 (11–30)	17 (11–26)	0.367	14.0 (11–22)	14 (11–21)	0.199
T-bil (mg/dL), median (IQR)	0.5 (0.4–0.6)	0.4 (0.4–0.6)	0.611	0.4 (0.3–0.6)	0.4 (0.3–0.5)	0.555
WBC (×10^3^/L), median (IQR)	6.6 (5.5–9.1)	6.9 (5.7–8.3)	0.732	7.6 (6.3–9.9)	7.3 (5.9–9.2)	0.482
Neutrophil (/L), median (IQR)	4,580 (3,660–6,482)	4,615 (3,449–8,473)	0.855	5,644 (4,593–8,878)	6,090 (4,678–8,723)	0.638
Hb (g/dL), median (IQR)	12.5 (11.5–13.4)	12.7 (11.4–14.0)	0.730	12.1 (10.5–13.0)	12.7 (11.6–13.7)	0.020
PLT (×10^4^/µL), median (IQR)	23.9 (18.7–30.4)	25.5 (20.1–35.9)	0.302	26.2 (21.2–35.9)	25.8 (20.0–33.0)	0.503
Histology, no. (%)	​	​	0.512	​	​	0.242
Adenocarcinoma	23 (74.2%)	45 (66.2%)	​	17 (34.7%)	24 (45.3%)	​
Squamous	4 (12.9%)	17 (25.0%)	​	27 (55.1%)	27 (50.9%)	​
Other	4 (12.9%)	6 (8.8%)	​	5 (10.2%)	2 (3.8%)	​
EGFR mutation positive, no. (%)	8 (25.8%)	19 (27.9%)	0.467	2 (4.1%)	8 (15.1%)	0.103
Chemotherapy regimen, no. (%)						
Combination with ICI	13 (41.9%)	35 (51.5%)	0.512	20 (40.8%)	10 (18.9%)	0.009
Combination with bevacizumab	18 (58.1%)	38 (55.9%)	1.000	1 (2.0%)	0 (0%)	0.480
CBDCA dose (mg), median (IQR)	480 (340–620)	450 (380–550)	0.972	430 (375–510)	440 (360–550)	0.471
PTX dose (mg), median (IQR)	270 (130–330)	275 (123–310)	0.975	​	​	​
Nab-PTX dose (mg), median (IQR)	​	​	​	160 (130–170)	160 (140–180)	0.958
History of radiation therapy, no. (%)	4 (12.9)	13 (19.1)	0.179	12 (24.5)	5 (9.4)	0.028
Line of therapy, no. (%)						
1st	21 (67.7%)	50 (73.5%)	1.000	46 (93.9%)	40 (75.5%)	0.014
2nd	6 (19.4%)	12 (17.6%)	0.778	3 (8.2%)	11 (20.8%)	<0.001

PTX, paclitaxel; nab-PTX, nab-paclitaxel; FosNTP, fosnetupitant; APR, aprepitant; IQR, interquartile range; Alb, albumin; AST, aspartate aminotransferase; ALT, alanine aminotransferase; T-Bil, total bilirubin; WBC, white blood cell; Hb, hemoglobin; PLT, platelet; EGFR, epidermal growth factor receptor; ICI, immune checkpoint inhibitor; CBDCA, carboplatin.

As our institution transitioned to a standardized antiemetic regimen incorporating FosNTP for the studied treatment protocols, no significant differences in patient demographics were observed regarding antiemetic medications between patients receiving either PTX or nab-PTX.

### 3.2 Frequency of adverse events in patients on PTX

The frequency of myelosuppression and liver dysfunction in patients using PTX is shown in Table 2.

**TABLE 2:** Incidence of adverse events in PTX-treated patients.

Adverse events	All grades		Grade ≥3			
	FosNTP (n = 31)	APR (n = 68)	FosNTP (n = 31)	APR (n = 68)	Or (95% CI)	p-value
Neutropenia	20 (64.5%)	34 (50.0%)	15 (48.4%)	18 (26.5%)	2.60 (1.07–6.32)	0.019
Anemia	26 (83.9%)	59 (86.8%)	2 (6.5%)	0 (0%)	-	-
Thrombocytopenia	16 (51.6%)	22 (32.4%)	1 (3.2%)	3 (4.4%)	0.722 (0.07–7.21)	0.412
Increased AST	4 (12.9%)	5 (7.4%)	0 (0%)	1 (1.5%)	-	-
Increased ALT	7 (22.6%)	13 (19.1%)	0 (0%)	0 (0%)	-	-
Increased T-Bil	3 (9.7%)	3 (4.4%)	0 (0%)	0 (0%)	-	-

OR, odds ratio; PTX, paclitaxel; FosNTP, fosnetupitant; APR, aprepitant; CI, confidence interval; AST, aspartate aminotransferase; ALT, alanine aminotransferase; T-Bil, total bilirubin.

Anemia was most frequently observed in patients using antiemetic agents (FosNTP: 83.9%, APR: 86.8%). Regardless of severity, patients using FosNTP tended to have more neutropenia (64.5% vs. 50.0%) and thrombocytopenia (51.6% vs. 32.4%) than did those using APR. Patients receiving FosNTP also demonstrated a higher incidence of grade ≥3 neutropenia throughout the entire treatment period (odds ratio [OR] = 2.60, 95% confidence interval [CI]: 1.07–6.32, p = 0.019). A trend toward more severe anemia in FosNTP-treated patients was evident throughout the entire treatment period (p = 0.096).

**FIGURE 2:**
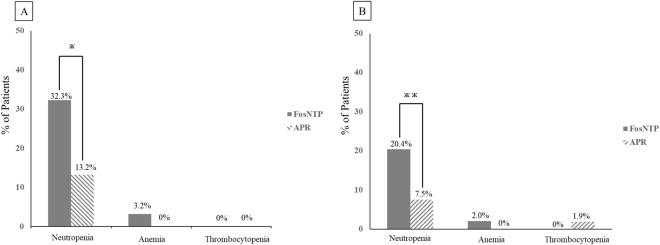
Incidence rate of grade ≥ 3 bone marrow suppression at the end of the first cycle **(A)** Patients receiving PTX; **(B)** Patients receiving nab-PTX. The incidence of myelosuppression in the FosNTP and APR groups was compared after the first cycle. FosNTP, fosnetupitant; APR, aprepitant; PTX, paclitaxel; nab-PTX, nanoparticle albumin-bound paclitaxel. ж *p* = 0.020. жж *p* = 0.040.

The incidence of grade ≥3 myelosuppression in PTX-treated patients at the end of the first cycle was compared between the FosNTP and APR groups (Figure 2A). At the conclusion of the first cycle, the incidence of grade ≥3 neutropenia was significantly higher in patients treated with FosNTP than in those treated with APR (p = 0.020). At the initiation of the second treatment cycle, PTX dose reductions were observed in seven patients (22.6%) in the FosNTP group and 10 patients (14.7%) in the APR group. However, no significant difference in the thrombocytopenia and anemia incidence was observed at the end of the first cycle. Additionally, one case of grade 3 AST elevation and one case of grade 4 thrombocytopenia were documented in patients receiving APR.

### 3.3 Frequency of adverse events in patients on Nab-PTX

Table 3 presents the frequency of adverse events in patients treated with nab-PTX. Anemia was the most frequent adverse event among patients receiving either antiemetic agent, regardless of the severity (FosNTP: 91.8%; APR: 90.6%). Over the entire treatment period, no significant difference was observed in the incidence of severe grade ≥3 myelosuppression.

**TABLE 3:** Incidence of adverse events in patients treated with nab-PTX.

Adverse events	All grades		Grade ≥3			
	FosNTP (n = 49)	APR (n = 53)	FosNTP (n = 49)	APR (n = 53)	Or (95% CI)	p-value
Neutropenia	26 (53.1%)	23 (43.4%)	10 (20.4%)	9 (17.0%)	1.25 (0.46–3.40)	0.182
Anemia	45 (91.8%)	48 (90.6%)	11 (22.4%)	7 (13.2%)	1.90 (0.67–5.38)	0.099
Thrombocytopenia	19 (38.8%)	25 (47.2%)	0 (0%)	1 (1.9%)	-	-
Increased AST	5 (10.2%)	2 (3.8%)	0 (0%)	1 (1.9%)	-	-
Increased ALT	10 (20.4%)	12 (22.6%)	1 (2.0%)	1 (1.9%)	1.08 (0.07–17.81)	0.504
Increased T-Bil	2 (4.1%)	1 (1.9%)	1 (2.0%)	0 (0%)	-	-

OR, odds ratio; nab-PTX, nab-paclitaxel; FosNTP, fosnetupitant; APR, aprepitant; CI, confidence interval; AST, aspartate aminotransferase; ALT, alanine aminotransferase; T-Bil, total bilirubin.

The incidence of grade ≥3 myelosuppression was compared at the conclusion of the first cycle (Figure 2B). At the end of the first cycle, the incidence of grade ≥3 neutropenia was significantly elevated in patients who received FosNTP (p = 0.040). At the initiation of the second treatment cycle, nab-PTX dose reductions were observed in 10 patients (20.4%) in the FosNTP group and four patients (7.5%) in the APR group. No significant differences in anemia or thrombocytopenia were observed between groups. Additional grade ≥3 adverse events included elevated ATL and total bilirubin levels in one case each in the FosNTP group and thrombocytopenia and elevated AST and alanine aminotransferase levels in one case each in the APR group.

### 3.4 Risk factor analysis for bone marrow suppression

Given the limited number of patients treated with FosNTP during the early post-approval period and the potential imbalance in baseline characteristics, multivariate logistic regression analyses were performed to identify independent risk factors for grade ≥3 neutropenia and anemia in the overall cohort. Baseline white blood cell and neutrophil counts were not incorporated due to insufficient variability, with >95% of patients presenting values within the normal range. As shown in Table 4, the use of FosNTP (OR = 2.27, 95% CI: 1.09–4.74, p = 0.029), male sex (OR = 3.91, 95% CI: 1.37–11.1, p = 0.011), and bevacizumab combination therapy (OR = 9.77, 95% CI: 3.12–30.53, p < 0.001) were identified as significant independent predictors of grade ≥3 neutropenia. In contrast, age, baseline albumin, and platelet count—although previously reported as risk factors—were not significantly associated with severe neutropenia in this study. Notably, the incidence of severe neutropenia did not differ significantly between PTX and nab-PTX regimens. Regarding grade ≥3 anemia, baseline grade 2 hemoglobin level (OR = 12.70, 95% CI: 2.83–57.05, p = 0.001) and the use of nab-PTX (OR = 9.99, 95% CI: 1.96–50.92, p = 0.006) emerged as significant predictors (Supplementary Table S3). Bevacizumab use was excluded from this model because no cases of severe anemia occurred in patients receiving bevacizumab. Grade ≥3 thrombocytopenia was not evaluated in multivariable analyses due to the limited number of observed events. In addition, the results of the multivariate analysis for Grade ≥3 neutropenia after excluding patients who received bevacizumab are presented in Supplementary Table S4. In this analysis, only baseline albumin <3.9 g/dL was identified as a significant risk factor (OR = 3.38, 95% CI = 1.32–8.66, p = 0.011).

**TABLE 4:** Multivariate analysis of risk factors for grade ≥3 neutropenia.

Variables	Multivariate analysis	
	Or (95% CI)	p-value
FosNTP use	2.27 (1.09–4.74)	0.029
Male	3.91 (1.37–11.10)	0.011
Bevacizumab	9.77 (3.12–30.53)	<0.001
Age ≥60	1.49 (0.52–4.24)	0.454
PTX	1.38 (0.50–3.79)	0.532
Immune checkpoint inhibitors	1.37 (0.62–3.05)	0.440
Baseline of alb <3.9 g/dL	2.05 (0.98–4.29)	0.056
Pre-existing grade2 anemia	3.35 (0.64–17.45)	0.152
Pre-existing grade 1 thrombocytopenia	2.17 (0.41–11.35)	0.360

OR, odds ratio; CI, confidence interval; FosNTP, fosnetupitant; PTX, paclitaxel; Alb, albumin.

## 4 Discussion

The prevention of chemotherapy-induced nausea and vomiting is crucial for maintaining patient quality of life. However, drug interactions between antiemetics and chemotherapeutic agents may increase the risk of adverse events such as myelosuppression, potentially leading to dose reduction or treatment interruption and compromising therapeutic efficacy. This study is the first to investigate the incidence of severe myelosuppression in patients with non-small cell lung cancer receiving the NK1 receptor antagonists FosNTP or APR in combination with PTX or nab-PTX, both of which are metabolized by CYP3A4.

Prior to chemotherapy, FosNTP is administered intravenously in conjunction with dexamethasone and 5-HT3 receptor antagonists. As an injectable formulation, it immediately achieves Tmax and undergoes rapid conversion to its active metabolite, NTP, which is subsequently distributed systemically (Tyler et al., 2022). In contrast, oral APR requires approximately 4 h to reach Tmax when administered 1–1.5 h before chemotherapy (Yang et al., 2020). In the present study, PTX was administered over 1–3 h depending on the dose, whereas nab-PTX was administered over 30 min. Consequently, APR remains in the absorption phase when PTX or nab-PTX administration commences, resulting in lower systemic concentrations (Yang et al., 2020). Given that the CYP3A4 inhibitory effect of NK1 receptor antagonists is concentration-dependent, FosNTP may exert a more pronounced effect than APR (Lanzarotti and Rossi, 2013).

Throughout the entire treatment course in patients treated with PTX, grade ≥3 neutropenia occurred in approximately 50% of FosNTP-treated patients, again representing a significant increase compared to the APR group. However, no differences in the anemia or thrombocytopenia incidence were observed between the groups. These findings indicate that medical staff should maintain vigilance for neutropenia, infection, and febrile neutropenia during the entire treatment course.

In patients receiving PTX, the incidence of grade ≥3 neutropenia following the first cycle was approximately 30% with FosNTP, representing a significant increase compared to APR. Of those included in the study, 66% of the patients received 175–200 mg/m^2^ PTX on day 1, resulting in substantial drug exposure concurrent with antiemetic administration. PTX exhibits nonlinear pharmacokinetics, partly attributable to excipients such as Cremophor EL and polyethylene glycol, which impede tissue distribution (Sparreboom et al., 1999; Salem et al., 2023). The duration of plasma PTX concentrations exceeding 0.05 μM correlates with neutropenia development (Gianni et al., 1995). Although the target diseases differed across studies, previous pharmacokinetic analyses have demonstrated that single-agent nab-PTX 260 mg/m^2^ and PTX 175 mg/m^2^ yield comparable systemic exposure per treatment cycle (20,324.49 ± 3,965.89 h·ng/mL for nab-PTX vs. 20,821.07 ± 5,391.37 h·ng/mL for PTX; p = 0.72) (Gardner et al., 2008). The same study also reported that plasma PTX concentrations declined to below the lower limit of quantification by 10 h after administration, indicating relatively rapid systemic elimination. In contrast, the duration of PTX infusion is known to substantially influence its pharmacokinetics. Compared with prolonged infusion, a 3-h infusion has been associated with significantly reduced clearance (Cl_3h_ = 42.8 ± 24.9 mL·h^-1^·m^-2^ vs. Cl_24h_ = 79.7 ± 24.3; p = 0.035), suggesting that shorter infusion schedules may increase exposure and thereby enhance toxicity (van Tellingen et al., 1999). However, these investigations evaluated PTX or nab-PTX as monotherapy, and pharmacokinetic profiles under coadministration with NK1 receptor antagonists remain uncertain. Among NK1 receptor antagonists, NTP has a markedly longer elimination half-life of approximately 70 h, far exceeding that of APR, which is approximately 10 h. Given this difference, it is plausible that NTP produces more sustained inhibition of CYP3A4, leading to reduced PTX clearance and enhanced myelosuppression (Abe et al., 2023). Moreover, PTX exhibits a high protein-binding rate of approximately 90%; therefore, hypoalbuminemia increases the unbound fraction of PTX and has been associated with augmented toxicity (Minami et al., 2006). Although this relationship has been described in gastric cancer, our study found no significant differences in serum albumin levels between groups, and albumin did not emerge as an independent risk factor in multivariable analysis (Nakazawa et al., 2019). These findings suggest that the intensified myelosuppression observed in our cohort is more likely attributable to prolonged CYP3A4 inhibition by NTP than to variations in albumin levels. Patient demographics, including antiemetic utilization, were also comparable between the groups, suggesting that antiemetic selection may influence neutropenia development.

Among nab-PTX recipients, no significant difference in the incidence of severe neutropenia between FosNTP and APR was observed throughout the entire treatment period. There was also no difference in the incidence of anemia or thrombocytopenia between FosNTP and APR. Previous clinical trials have reported that PTX is associated with a higher incidence of severe neutropenia than nab-PTX, whereas nab-PTX demonstrates a greater propensity for severe anemia and peripheral neuropathy than PTX (Gradishar et al., 2005; Socinski et al., 2012). In this study, the higher anemia incidence in nab-PTX recipients aligns with these previous findings. In this study, a difference in baseline hemoglobin levels at treatment initiation was observed between the APR and FosNTP groups in patients receiving nab-PTX, which may have influenced the results; therefore, further investigation is warranted.

Multivariate analysis identified the use of FosNTP user, male sex, and concomitant bevacizumab therapy as independent risk factors for grade ≥ 3 neutropenia. The finding that FosNTP use may contribute to neutropenia suggests a potential pharmacological influence that warrants further investigation. Additionally, the extraction of male sex as a significant risk factor implies that multifactorial mechanisms—such as differences in body fat composition, hepatic metabolic enzyme activity, and sex-related variations in drug distribution volume—may be involved. However, prior studies have reported inconsistent conclusions regarding sex-based differences, indicating the need for continued evaluation from both pharmacological and sex-specific perspectives. Furthermore, the increased incidence of neutropenia in patients receiving anti-angiogenic therapy is consistent with previous reports demonstrating severe myelotoxicity in regimens combining docetaxel and ramucirumab (Yoh et al., 2016). Since anti–vascular endothelial growth factor activity has been implicated in alterations to the hematopoietic microenvironment, the present findings likely reflect the clinical relevance of such biological effects. Given that neutropenia was frequently observed among patients treated with bevacizumab, the potential impact of the treatment regimen itself cannot be excluded. In this study, a high incidence of Grade ≥3 neutropenia was observed among patients receiving paclitaxel, and approximately half of these patients were also treated with bevacizumab. Therefore, in the multivariate analysis conducted after excluding bevacizumab-treated patients, the reduced sample size may have resulted in insufficient statistical power to adequately evaluate the association with FosNTP. Notably, more than 95% of the patients in this study exhibited normal baseline leukocyte and neutrophil counts at treatment initiation. Thus, individuals with impaired bone marrow reserve prior to therapy may be at even higher risk of developing severe neutropenia. In contrast, for grade ≥ 3 anemia, pre-existing grade 2 anemia and the use of nab-PTX emerged as significant risk factor. The results of multivariate analysis also suggested that nab-PTX was significantly associated with severe anemia, consistent with previous reports (Gradishar et al., 2005; Socinski et al., 2012). This finding underscores the importance of hematologic assessment before initiating chemotherapy and highlights the need for early supportive interventions or careful consideration of treatment delays. Because our observations were derived from a single-center Japanese cohort, the potential influence of genetic variability cannot be excluded, particularly if CYP-mediated mechanisms contributed to the increased incidence of adverse events, as hypothesized. Therefore, further investigations incorporating CYP3A4 genetic polymorphisms and including diverse ethnic populations will be essential for a more comprehensive evaluation.

A difference in the incidence of grade ≥3 neutropenia between the FosNTP and APR groups was detected after the first treatment cycle. One potential explanation for this phenomenon involves the influence of the formulation characteristics. Nab-PTX nanoparticles are approximately 130 nm in diameter, which rapidly dissociate following administration (Spada et al., 2021; Sparreboom et al., 2005). A previous report comparing intravenous administration of nab-PTX at 260 mg/m^2^ over 30 min and conventional PTX formulations at 175 mg/m^2^ over 180 min showed that while the areas under the curve were similar, the volume of distribution was significantly higher for nab-PTX, indicating enhanced tissue penetration (Desai et al., 2006; Spada et al., 2021; Sparreboom et al., 2005). This suggests that the rapid tissue distribution of nab-PTX may have reduced its interaction time with CYP3A4 in the plasma, thereby further attenuating the inhibitory effect. Moreover, given that nab-PTX was administered using a dose-divided schedule (days 1, 8, and 15), the amount of drug per administration was relatively lower compared to PTX (Socinski et al., 2018; West et al., 2019). Since CYP3A4 inhibition is concentration-dependent, the reduced systemic exposure to nab-PTX at any given time likely minimized the impact of FosNTP-mediated CYP3A4 inhibition. In addition, dose modifications or treatment interruptions of nab-PTX were implemented based on interim laboratory results and patient conditions, which may have contributed to the absence of significant differences in the incidence of adverse events throughout the treatment period. Nonetheless, the higher frequency of PTX dose reductions observed in the FosNTP group compared with the APR group may reflect differences in treatment tolerability between the two antiemetic regimens. However, given the limited sample size and potential influence of patient-specific factors, these findings should be interpreted with caution.

Although liver function parameters (AST, alanine aminotransferase, and total bilirubin) were examined, no significant alterations were observed with the antiemetic transitions. One potential explanation is that these adverse events demonstrate a low incidence, even in clinical trials, and the sample size in the present study was insufficient for adequate evaluation of these outcomes (Socinski et al., 2018; West et al., 2019).

This study has some limitations. First, this was a retrospective analysis conducted at a single institution with a limited sample size. As FosNTP has only recently become available in clinical practice, the current number of reported cases remains substantially lower than that of APR. Additionally, due to the implementation of subgroup analyses in patients receiving PTX and nab-PTX, the number of cases in each group was limited. Therefore, we cannot rule out the possibility that the small sample size in the FosNTP group may have influenced the incidence of adverse events observed in this cohort. In addition, as the FosNTP group was introduced during the later period of the study, the possibility of temporal bias cannot be excluded. Furthermore, the multivariate analysis may have been affected by the limited sample size, which restricted our ability to fully evaluate the impact of factors such as bevacizumab use. Second, our analysis was restricted to myelosuppression and hepatotoxicity. Peripheral neuropathy, myalgia, and fatigue—well-recognized adverse effects of taxane therapies—were excluded because the subjective nature of these symptoms and incomplete documentation in the retrospective dataset rendered reliable grading infeasible. Consequently, we were unable to assess the potential influence of antiemetic agents on these clinically relevant toxicities. Given their substantial impact on treatment continuity, we plan to evaluate these adverse events in future prospective studies using standardized patient-reported outcome measures. Third, because this study was retrospective, plasma concentrations of PTX and nab-PTX were not measured, precluding detailed pharmacokinetic analyses. Our study was conducted under the hypothesis that CYP3A4-mediated drug–drug interactions induced by antiemetic agents contributed to the increased incidence of adverse events such as neutropenia, based on prior reports. However, whether the observed findings directly reflect the mechanisms we hypothesized remains uncertain and warrants further investigation. To address this, we aim to conduct prospective studies that include comprehensive assessments of additional toxicities, such as peripheral neuropathy, along with pharmacokinetic analyses. Ultimately, patients receiving concomitant medications with moderate-to-strong inhibitory or inductive effects on CYP3A4 at treatment initiation were excluded from the analysis. However, due to the nature of retrospective studies, we cannot rule out the possibility that some patients may have initiated such medications at outside facilities during treatment, making detailed tracking of medication histories challenging. Numerous drugs, including azole antifungals and rifampicin, are known to inhibit or induce CYP3A4 activity. Therefore, the possibility that some patients initiated these agents at other institutions during treatment cannot be excluded. As our findings were derived from a single-center Japanese cohort, the potential influence of CYP3A4 genetic polymorphisms and interethnic differences cannot be excluded if CYP-mediated mechanisms contribute to toxicity. Therefore, further studies incorporating CYP3A4 genotyping and analyses across diverse populations are warranted. Moreover, ongoing pharmacovigilance and real-world data will be crucial to determine the durability and generalizability of these observations across broader clinical settings. Taken together, these limitations indicate that our preliminary findings should be interpreted with caution and underscore the need for validation in future studies.

In conclusion, our investigation suggests that FosNTP utilization in PTX-containing regimens may increase the incidence of grade ≥3 neutropenia compared to APR, underscoring the importance of enhanced monitoring throughout the treatment period. Furthermore, multivariate analysis also identified the use of FosNTP as a risk factor for severe neutropenia. Anorexia and nausea represent the most distressing adverse events experienced by cancer patients. Consequently, the sustained antiemetic efficacy of NK1 receptor antagonists can directly influence daily functioning and substantially improve quality of life. However, our findings also indicate the potential for severe neutropenia resulting from the interaction between FosNTP and PTX. Although peg-G-CSF is considered effective for preventing severe neutropenia, its substantial cost necessitates careful consideration of the associated financial burden in clinical practice (Hata et al., 2022). These findings collectively underscore the importance of selecting antiemetic agents based on the specific chemotherapeutic regimen employed. To validate and expand these preliminary findings, we plan to conduct prospective studies with larger sample sizes, a broader range of drug classes, and integrated pharmacokinetic analyses.

## Data Availability

The raw data supporting the conclusions of this article will be made available by the authors, without undue reservation.
