# Supplementary material for: The impact of NK1 receptor antagonist selection on chemotherapy-related adverse events: a retrospective study of adverse events in paclitaxel or nab-paclitaxel and carboplatin combination therapy with fosnetupitant and aprepitant use
Source: Front Pharmacol. 2026 May 11;17:1712949. doi: 10.3389/fphar.2026.1712949 (PMC13199330; doi:10.3389/fphar.2026.1712949)
Supplement: Supplementary file 1 [file Supplementaryfile1.docx]

Supplementary table1. Primary administration schedule for the PTX+CBDCA regimen

|  | Day 1 |
| --- | --- |
| Dexamethasone　 16.5mg  *d*-Chlorpheniramine 5mg  Famotidine 20mg  FosNTP　　 235mg  Palonosetron　　　　 0.75mg  Intravenous infusion 30 min | **↓** |
| Physiological saline　 100mL  Intravenous infusion 30 min | **↓** |
| PTX　 　　　 175mg/m^2^  Intravenous infusion 180 min | **↓** |
| CBDCA 　AUC(4~6)  Intravenous infusion 60 min | **↓** |

PTX, paclitaxel; CBDCA, Carboplatin; FosNTP, fosnetupitant; APR, aprepitant; nab-PTX, nanoparticle albumin-bound paclitaxel.

When APR was administered orally instead of FosNTP, it was given 1 to 1.5 h before anticancer drug administration. When immune checkpoint inhibitors were used, they were administered over 30 min before antiemetic administration on Day 1. For bevacizumab-containing regimens, bevacizumab was administered over 30 min after the Day 1 antiemetic administration, followed by nab-PTX. In regimens in which PTX was administered on Days 1, 8, and 15, antiemetic agents and normal saline were administered prior to PTX infusion, which was subsequently delivered over 60 min. Additionally, on Days 2–3, oral dexamethasone tablets (4–8 mg) were administered for prophylactic antiemesis.

Supplementary table 2. Primary Administration Schedule for the nab-PTX+CBDCA Regimen.

|  | Day 1 | Day 8 | Day 15 |
| --- | --- | --- | --- |
| Dexamethasone 6.6mg  FosNTP 　　 235mg  Palonosetron　　　　 0.75mg  Intravenous infusion 30 min | **↓** |  |  |
| Dexamethasone　 6.6mg  Intravenous infusion 30 min |  | **↓** | **↓** |
| nab-PTX　 100mg/m^2^  Intravenous infusion 30 min | **↓** | **↓** | **↓** |
| CBDCA AUC(4~6)  Intravenous infusion 60 min | **↓** |  |  |

nab-PTX, nanoparticle albumin-bound paclitaxe; CBDCA, Carboplatin; FosNTP, fosnetupitant; APR, aprepitant;

When APR was administered orally instead of FosNTP, it was given 1 to 1.5 h before anticancer drug administration. When immune checkpoint inhibitors were administered, they were given over 30 min before antiemetic administration on Day 1. For bevacizumab-containing regimens, bevacizumab was administered over 30 min after the Day 1 antiemetic administration, followed by nab-PTX. Additionally, on Days 2–3, oral dexamethasone tablets (4–8 mg) were administered for prophylactic antiemesis.

Supplementary table 3. Multivariate analysis of risk factors for grade 3 or higher anemia.

| Variables | Multivariate analysis | |
| --- | --- | --- |
|  | OR (95% CI) | *P*-value |
| Pre-existing grade2 anemia | 12.70 (2.83-57.05) | 0.001 |
| Nab-PTX | 9.99 (1.96-50.92) | 0.006 |
| FosNTP | 2.30 (0.74-7.19) | 0.151 |
| Men | 6.50 (0.69-61.30) | 0.102 |
| Age ≥60 | 2.36 (0.35-16.05) | 0.380 |
| Immune checkpoint inhibitors | 1.30 (0.38-4.49) | 0.679 |
| Base line of ALB <3.9 | 1.30 (0.22-2.66) | 0.679 |
| Pre-existing grade1 thrombocytopenia | 3.01 (0.35-25.78) | 0.314 |

OR, odds ratio; CI, confidence interval; FosNTP, fosnetupitant; nab-PTX, nanoparticle albumin-bound paclitaxel; Alb, albumin.

Supplementary table 4. Multivariate analysis of risk factors for grade 3 or higher neutropenia excluding patients receiving bevacizumab.

| Variables | Multivariate analysis | |
| --- | --- | --- |
|  | OR (95% CI) | p-value |
| FosNTP use | 2.29 (0.89–5.88) | 0.084 |
| Baseline of Alb < 3.9 g/dL | 3.38 (1.32–8.66) | 0.011 |
| Age ≥ 60 | 3.50 (0.40–30.77) | 0.259 |
| Immune checkpoint inhibitors | 1.25 (0.42–3.72) | 0.630 |
| PTX | 1.25 (0.27–3.55) | 0.640 |
| Pre-existing grade2 anemia | 3.51 (0.39–31.63) | 0.263 |
| Pre-existing grade 1 thrombocytopenia | 2.89 (0.44–19.18) | 0.272 |

OR, odds ratio; CI, confidence interval; FosNTP, fosnetupitant; PTX, paclitaxel; Alb, albumin.

Patients receiving paclitaxel were excluded from the analysis because all of them were concomitantly treated with bevacizumab, making it difficult to distinguish the independent effect of paclitaxel.
